# Effects of high-pressure homogenization and enzymatic hydrolysis on the physicochemical properties of gelatin-stabilized tuna oil-based emulsion

**DOI:** 10.1016/j.fochx.2026.103977

**Published:** 2026-05-12

**Authors:** Junyong Xuan, Imad Khan, Huilan Zeng, Zehua Qiu, Zongyuan Han, Zefu Wang, Shucheng Liu, Qiuyu Xia

**Affiliations:** a College of Food Science and Technology, Guangdong Ocean University, Guangdong Provincial Key Laboratory of Aquatic Product Processing and Safety, Guangdong Province Engineering Laboratory for Marine Biological Products, Guangdong Provincial Engineering Technology Research Center of Seafood, Guangdong Provincial Engineering Technology Research Center of Prefabricated Seafood Processing and Quality Control, Zhanjiang 524088, China; b Collaborative Innovation Center of Seafood Deep Processing, Dalian Polytechnic University, Dalian 116034, China

**Keywords:** High-pressure homogenization, Tuna oil, Hydrolyzed acylglycerol, Emulsion, Interfacial properties

## Abstract

This study investigated the modification effects of high-pressure homogenization (HPH) combined with enzymatic hydrolysis of fish oil on gelatin-stabilized emulsions. Emulsions were prepared using tuna oil and acylglycerol (CALA-ACY) derived from its hydrolysis by *Candida antarctica* lipase as the oil phases. These emulsions were then subjected to HPH at 60 MPa for three cycles. Results showed that HPH treatment enhanced the emulsification properties of emulsions. CALA-ACY-based emulsions exhibited decreased surface charge and interfacial protein adsorption. Notably, the synergistic effect of HPH and enzymatic hydrolysis of the oil phase reduced the β-sheet structure content while enhancing the random coil structure content of proteins in the emulsion. At the same time, the decrease in surface charge and apparent viscosity to their lowest values resulted in reduced long-term storage stability of the emulsion. This study provided a theoretical basis for the utilization of fish oil acylglycerol-based emulsions and the promotion of HPH technology

## Highlights

• Desirable amount of diacylglycerol (DAG) was produced via enzymatic modification.
• Competitive adsorption between DAG and gelatin occurred at the oil–water interface.
• Gelatin conformation of emulsion was transformed by DAG and HPH treatment.
• HPH treatment exacerbated the adsorption of DAG at the oil–water interface.
• Both HPH treatment and DAG adsorption weaken the storage stability of emulsions.

## 1 Introduction

Fish oil is widely recognized as a functional food ingredient because it is an abundant source of naturally occurring omega-3 polyunsaturated fatty acids (ω-3 PUFAs), including eicosapentaenoic acid (EPA) and docosahexaenoic acid (DHA). Recent research has confirmed the health benefits of these fatty acids, including reducing cardiovascular disease risk, regulating inflammatory responses, and improving brain function (Li, Ning, et al., 2025; Wei et al., 2025). However, fish oil is particularly vulnerable to oxidation and deterioration, hence biopolymer-based stabilizing procedures are commonly used to extend its shelf life.

Protein emulsions constitute a fundamental method for oil encapsulation. Gelatin, an affordable and accessible amphiphilic protein, has superior gelling, water-retention, and emulsifying characteristics, interpreting it extensively utilized in the food industry (Shi et al., 2025). Protein-stabilized emulsions, including those prepared with gelatin, can be served as well-defined model systems for investigating the fundamental interfacial phenomena that govern emulsion performance, as they allow for systematic examination of how processing technology and parameters influence protein adsorption behavior and droplet interfacial properties (Liu et al., 2024). This has prompted extensive research into efficient emulsion homogenization processes and the identification of high-performance emulsifiers to achieve synergistic stabilization. High-pressure homogenization (HPH) represents an advanced emulsion processing technology that has been employed to modulate the quality of protein-stabilized emulsions. The substantial mechanical energy imparted by HPH, in combination with precisely engineered homogenizing valves, enables coarse emulsions to undergo intense shear forces and high-frequency collisions, resulting in the formation of finely dispersed and uniform emulsions (Li et al., 2024). Consistent with this, Zhao et al. (2025) reported that, compared with high-speed shear homogenization at 10,000 rpm, two cycles of HPH treatment at 25 MPa significantly reduced particle size and enhanced the stability of peanut oleosome emulsions, thereby improving their resistance to stratification and flocculation.

It is widely recognized that the composition of oil phase and its interactions with proteins are fundamental factors affecting the characteristics of protein-stabilized emulsions (Petelin et al., 2025; Zhang et al., 2025). Lipase-catalyzed hydrolysis of fish oil serves as an effective modification strategy to alter specific fatty acid distributions, enabling the generation of structured acylglycerols with improved emulsifying capacity and nutritional value (Chen, Liu, et al., 2023; Xie et al., 2025). Key properties, including the lipid composition of oil phases and the distribution of specific fatty acids on the glycerol backbone, may participate in regulating protein adsorption behavior at the oil–water (O—W) interface. However, studies examining the distinct effects of HPH on the interfacial properties and associated interaction forces of emulsions prepared before and after fish oil hydrolysis remain limited.

**Fig. 1:**
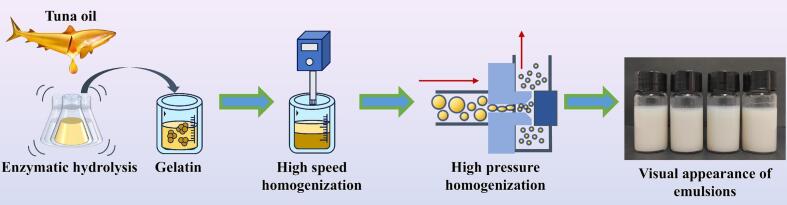
Schematic illustration of synergistic effect of high pressure homogenization and enzymatical hydrolysis on the physicochemical properties of gelatin-stabilized emulsion.

In the present work, *Candida antarctica lipase* (CALA) was used to hydrolyze tuna oil to generate acylglycerols with a modified lipid composition. Subsequently, distinct gelatin-stabilized O/W emulsions were prepared using high-speed homogenization and HPH techniques, respectively (Fig.1). By comparing the effects of HPH and the enzymatic hydrolysis of oil phase on the physicochemical properties of the emulsions, particularly their interfacial characteristics. The synergistic regulatory role of processing methods and lipid composition of oil phases on emulsion properties was elucidated. The findings of this study offered new insights into the preparation process and quality control strategies for fish oil-based emulsions.

## 2 Materials and methods

### 2.1 Materials

Tuna oil was purchased from Nu-Mega Co., Ltd. (Melbourne, Victoria, Australia). Lipase CALA with an enzyme activity of 6000 U/g, was purchased from Novozymes Co., Ltd. Gelatin from Porcine skin (Vetec™ reagent, Type A, approximately 300 g Bloom) was purchased from Sigma-Aldrich Co., Ltd. All other chemicals were purchased and used were of analytical grade.

### 2.2 Preparation of hydrolyzed acylglycerol

Tuna oil was mixed with 5% (*w*/w) lipase CALA and a 1.8-fold (*v*/*w*) phosphate buffer at pH 7.0, purged with nitrogen, and stirred at 400 rpm for 6 h. A specified volume of 0.5 M KOH (in a 30% ethanol solution) was introduced to terminate the reaction and neutralize free fatty acids generated during hydrolysis. Hydrolyzed acylglycerol was extracted using n-hexane, and the organic solvent was removed using rotary evaporation at 40 °C. The residual organic solvent was eliminated using nitrogen to obtain the hydrolyzed acylglycerol sample, named CALA-ACY.

### 2.3 Lipid profile analysis of tuna oil and its hydrolyzed acylglycerol

Methyl esterification of oil samples was adopted following our previously published protocol (Xuan et al., 2022). Fatty acid composition was analyzed using a TQ8040NX gas chromatography-mass spectrometer (Shimadzu Co., Ltd., Japan) equipped with an Inert Cap Pure-WAX silica capillary column (30 m × 0.25 mm, 0.25 μm).

Briefly, a 10 μL of oil sample was dissolved in 4 mL of n-heptane. Apply 1 μL of mixture was then dispensed onto the plate and developed in a solvent chamber containing n-hexane/diethanolamine/formic acid (60,17,0.2, *v*/v/v) for 20 min. The relative contents of triacylglycerol (TAG), diacylglycerol (DAG), and monoacylglycerol (MAG) in oil samples were analyzed using thin-layer chromatography (SF-2020 system, Shandong Zibo Shanfen Analysis Instrument Co., Ltd., Zibo, Shandong, China) equipped with an MD-FID detector.

### 2.4 Preparation of emulsions

12 g of gelatin was incorporated into138 g of ultrapure water to produce an 8% (*w*/w) suspension. Then, it was place in a 50 °C water bath until the gelatin was entirely dissolved, resulting in a clear, transparent solution is formed. Subsequently, 20 g of tuna oil or CALA-ACY sample was added to the gelatin solution and pre-emulsified for 5 min using a digital overhead stirrer at 1200 rpm. Following that, further emulsification was performed using a WIGGENS O-5000 high-speed shear homogenizer at 15,000 rpm for 15 min. To prevent sample overheating, emulsification was performed with 1-min intervals every 5 min. The resulting samples were designated Emulsion–tuna oil and Emulsion–CALA-ACY, respectively. Subsequently, HPH was carried out using a high-pressure homogenizer (GJB30, Super Homogeneous Pump Co., Ltd., Changzhou, China) equipped with a cooling water circulation system. The processing condition of HPH was 60 MPa for 3 cycles. And the treated samples were designated HPH–Emulsion–tuna oil and HPH–Emulsion–CALA-ACY, respectively.

### 2.5 Microstructure observation of emulsions

Fresh emulsion was diluted with an equal volume of ultrapure water and the microstructure was observed using an optical microscope equipped with a camera (OLYMPUS CX43, Oberkochen, Baden-Württemberg, Germany) in brightfield mode.

### 2.6 Analysis of interfacial tension

A droplet of fish oil was introduced into the gelatin solution using a syringe. The interfacial tension between different lipid samples and the gelatin solution was then measured using a contact angle/surface tensiometer (LSA100, Lauda Scientific, Lauda-Königshofen, Germany). The specific interfacial tension values were calculated using the Young-Laplace equation.

### 2.7 Emulsifying activity index (EAI) and emulsifying stability index (ESI) analysis

Freshly prepared emulsion was diluted 100-fold with a 0.1% sodium dodecyl sulfate solution. Absorbance of emulsions was determined at 500 nm using a UV–Vis spectrophotometer (UV-2550, Shimadzu, Co., Ltd., Kyoto, Japan). Absorbance of emulsions was reexamined after a duration of 12 h. The Eqs. (1), (2) for calculating EAI (m^2^/g) and ESI (%) are as follows:

(1) $$\mathrm{EAI}\left(\frac{m^{2}}{g}\right)=\frac{2\times 2.303\times A_{0}\times D}{C\times \left(1-\varphi \right)\times 10000}$$

(2) $$\mathrm{ESI}\left(\%\right)=\frac{A_{12}}{A_{0}}\times 100$$

In Eqs. (1), (2), A₀ denotes the absorbance at 500 nm at 0 min. A₁₂ denotes the absorbance at 500 nm at 12 h. D represents the dilution factor. C indicates the mass fraction of protein (g/mL), while φ signifies the volume fraction of the oil phase.

### 2.8 Analysis of average particle size and zeta potential of emulsions

Following a 400-fold dilution of emulsions with ultrapure water, the particle size and zeta potential were determined using a NanoZS Zetasizer (Malvern Instruments Ltd., Worcestershire, UK) at 25 °C. The refractive indices of the dispersed phase and the continuous phase were set to 1.467 and 1.330, respectively. And the absorption index of the dispersed phase was 0.01.

### 2.9 Analysis of adsorbed protein concentration (AP) of emulsions

The interfacial protein adsorption of emulsions was determined using the method of Cao et al. (2022) with slight modifications. Following centrifugation of emulsions at 4 °C and 10,000 rpm for 20 min, the transparent supernatant layer was collected using a syringe, ensuring the exclusion of oil droplets and large protein aggregates. The supernatant was then filtered through a 0.45 μm membrane to remove residual impurities. The initial protein concentration of emulsions and the protein concentration in the supernatant, which was not adsorbed at the O—W interface, were determined using the Lowry method, which involved measuring the absorbance of the sample at 750 nm using a UV–Vis spectrophotometer (UV-2550, Shimadzu, Co., Ltd., Kyoto, Japan). AP(%) was calculated using Eq. (3):

(3) $$\mathrm{AP}\left(\%\right)=\frac{C_{\mathrm{INI}}-C_{\mathrm{SER}}}{C_{\mathrm{INI}}}\times 100$$

C_INI_ represents the initial protein concentration in the emulsion (mg/mL), while C_SER_ represents the protein concentration in the supernatant after centrifugation that was not adsorbed at the O—W interface (mg/mL).

### 2.10 Analysis of rheological properties of emulsions

The rheological properties of the emulsions were determined using a rheometer (HAAKE MARS III, Thermo Fisher Scientific, Shanghai, China) equipped with a P35 probe and a 0.5 mm measurement gap. Approximately 1 mL of fresh emulsion was deposited onto a flat plate. Following a 5-min equilibration, the apparent viscosity of the emulsion was measured at 25 °C under shear rates ranging from 0.1 to 100 s^−1^. Subsequently, a strain sweep at 10 rad/s was conducted throughout the strain range of 0.1–10% strain to determine the linear viscoelastic region. The storage modulus (G′) and loss modulus (G″) were determined by oscillatory frequency scanning ranging from 0.1 to 15 Hz with a 1% strain applied.

### 2.11 Analysis of protein conformation

The secondary structure of protein in the emulsions was analyzed using a circular dichroism (CD) spectrometer (Chirascan V10, Applied Photophysics Ltd., Leatherhead, England). The scanning wavelength was established at 180–260 nm, and the speed was set to 100 nm/min. The tertiary structure of protein in the emulsions was determined using a fluorescence spectrometer (RF-5301pc, BRUKER, Karlsruhe, Germany). The emission slit width and increment were designated as 5 nm and 10 nm, respectively. The excitation wavelength was established at 280 nm, while the emission wavelength ranged from 300 to 400 nm.

### 2.12 Analysis of surface hydrophobicity of emulsions

A 20 μL of 8 mM 1-anilino-8-naphthalenesulfonic acid fluorescent probe was introduced into 4 mL of emulsion (diluted to concentrations of 62.5-, 125-, 250-, 500-, and 1000-fold, respectively). Subsequently, they were mixed thoroughly and allowed to react under light-protected condition for 30 min. Following that, the fluorescence intensity was quantified using a fluorescence spectrometer (RF-5301pc, BRUKER, Karlsruhe, Germany). The excitation and emission wavelengths were established at 390 nm and 470 nm, respectively. The relative fluorescence intensity (RFI) for each concentration was calculated using Eq. (4):

(4) $$\mathrm{RFI}=\frac{F_{m}-F_{0}}{F_{0}}$$

Among these, F_m_ and F_0_ represent the fluorescence intensity of the protein–ANS complex and that of ultrapure water, respectively. Surface hydrophobicity is quantified as the initial slope of RFI versus protein concentration (mg/mL).

### 2.13 Analysis of creaming index (CI) of emulsions

Fresh emulsions were added to sample bottles and stored at 25 °C for 15 days. Consistently record the separation of the supernatant. The CI of emulsions was calculated using Eq. (5):

(5) $$\mathrm{CI}\left(\%\right)=\frac{H_{S}}{H_{T}}\times 100$$

Where H_S_ denotes the height of the lower clear liquid layer in centimeters (cm), whereas H_T_ denotes the total height of the entire emulsion in centimeters (cm).

### 2.14 Analysis of peroxide value (POV) and thiobarbituric acid reactive substances (TBARS) of emulsions

The formation of primary and secondary oxidation products during storage was determined using the previous method (Jiang et al., 2022) with minor modifications.

For the POV, 3 mL of emulsion was mixed with 15 mL of isooctane/2-isopropanol (3,1, *v*/v). The mixture was then centrifuged at 9000 rpm for 3 min, and the organic supernatant was collected. Subsequently, 28 mL of methanol/1-butanol (2,1, v/v), 2 mL of the organic supernatant, and 300 μL of a freshly prepared mixture containing equal volumes of 3.94 M ammonium thiocyanate and Fe^2+^ solution were added to a centrifuge tube. After the vortex of 20 min, the absorbance was measured at 510 nm using a UV–Vis spectrophotometer (UV-2550, Shimadzu, Co., Ltd., Kyoto, Japan). The peroxide concentration in the samples was calculated based on a standard curve prepared using cumene hydroperoxide.

For the TBARS value, 3 mL of emulsion was mixed with 4.5 mL of ultrapure water, followed by the addition of 3 mL of TBA-TCA reagent (containing 15% trichloroacetic acid, 2% hydrochloric acid, and 0.375% 2-thiobarbituric acid). The mixture was heated in a boiling water bath for 15 min and then cooled at 25 °C for 10 min. The resulting solution was filtered through a 0.55 μm aqueous phase filter membrane, and the absorbance of the filtrate was measured at 532 nm using a UV–Vis spectrophotometer (UV-2550, Shimadzu, Co., Ltd., Kyoto, Japan).

### 2.15 Statistical analysis

Results are conducted as “mean ± standard deviation” from three independent experimental replicates. A one-way analysis of variance was conducted using IBM SPSS Statistics 26 (IBM Corporation, Armonk, NY, USA). A *p* < 0.05 was considered statistically significant. All figures in this paper were created using Origin 2021 software.

## 3 Results and discussion

### 3.1 Lipid profile analysis of tuna oil and its hydrolyzed acylglycerol

**Fig. 2:**
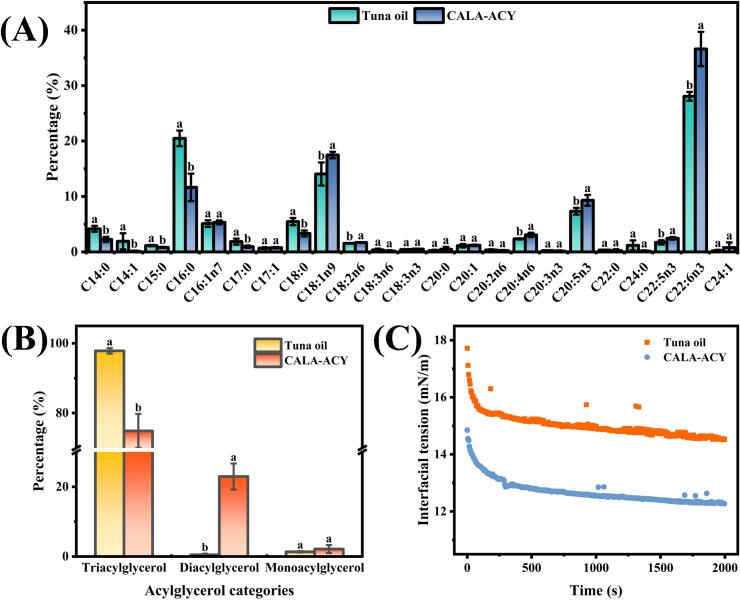
Analysis of lipid profile of tuna oil and its hydrolyzed acylglycerol. (A) Fatty acid composition. (B) Lipid class. (C) Interfacial tension.

Fig. 2A displays the content of fatty acids in two oil samples. Following hydrolysis by lipase CALA, the content of saturated fatty acids represented, particularly C14:0, C16:0, and C18:0 in tuna oil decreased dramatically (*p* < 0.05). In contrast, the total content of ω-3 PUFAs, including C20:5n3, C22:5n3, and C22:6n3 rose from 37.15% to 48.40%. Additionally, the content of C18:1n9 exhibited a notable increase. The lipid class of tuna oil and its hydrolyzed acylglycerol is illustrated in Fig. 2B. TAG constituted the predominant lipid in tuna oil, accounting for nearly 100%. In the presence of lipase CALA, a fraction of TAGs was hydrolyzed into DAGs and MAGs. Notably, the content of DAG exhibited an impressive alteration, rising from 0.51% to 22.96%. In comparison, the content of MAG showed no significant variation (*p* > 0.05). This discrepancy may arise from the differences in hydrolysis equilibrium constants, leading to TAGs more readily release fatty acids compared to DAGs (Morales-Medina et al., 2018). The interfacial tension between tuna oil as well as hydrolyzed acylglycerol and gelatin is depicted in Fig. 2C. Throughout the complete measurement range of 0–2000 s, both two oil samples demonstrated time-dependent variations in interfacial tension; however, hydrolyzed acylglycerol consistently displayed lower interfacial tension value than tuna oil. This phenomenon could be attributed to the rapid adsorption behavior of DAGs with strong surface activity at the O—W interface (Xuan et al., 2024). Thus, enzymatic hydrolysis improved the lipid profile of tuna oil, producing structured lipids with optimum polyunsaturated fatty acid ratios and DAG accumulation. This product showed potential as a novel, health-promoting emulsifier for application in the food industry.

### 3.2 Characterization of emulsions

**Fig. 3:**
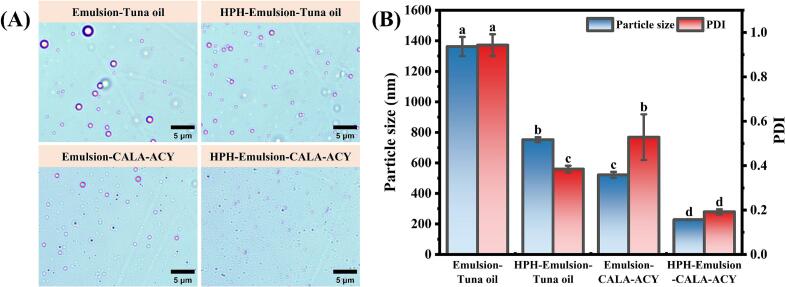
(A) Microstructural morphologies of emulsions based on tuna oil and CALA-ACY before and after HPH. The magnification of optical microscope was 100× and the bar is 5 μm. (B) Particle size and PDI of different emulsions.

Emulsion-tuna oil and emulsion-CALA-ACY were developed using tuna oil and its hydrolyzed acylglycerol as the oil phases, employing high-speed homogenization. These emulsions were further homogenized at 60 MPa for three cycles to prepare HPH-emulsion-tuna oil and HPH-emulsion-CALA-ACY, respectively. The microstructures of all four emulsions are displayed in Fig. 3A. As shown in Fig. 3B, in contrast to the emulsions obtained by high-speed homogenization, the particle size and PDI of emulsions treated by HPH were considerably reduced (*p* < 0.05). This is due to the high-speed turbulence and intense shear forces generated by HPH facilitated the formation of smaller and more uniformly dispersed droplets (Chen et al., 2025). Upon transitioning the oil phase from tuna oil to CALA-ACY, the particle size of emulsion significantly decreased from 1362.33 nm to 521.70 nm (*p* < 0.05). This can be ascribed to the emulsifying capacity of DAGs in the oil phase (Liu, Lee, et al., 2025). Moreover, compared to other three emulsions, HPH-emulsion-CALA-ACY exhibited the smallest particle size (228.57 nm) and PDI (0.19), indicating that the combination of HPH with enzymatic hydrolysis of oil phase facilitated the formation of nanoemulsions with excellent features.

### 3.3 Analysis of interfacial properties of emulsions

**Fig. 4:**
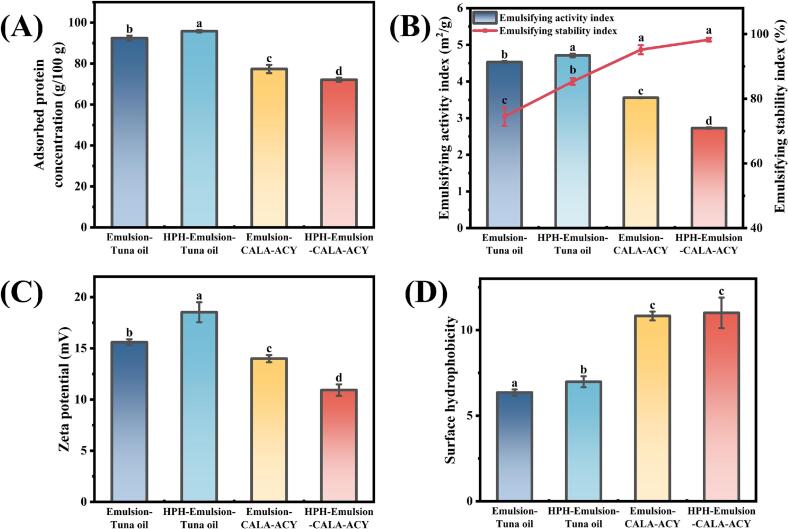
Characterization of emulsions of tuna oil and CALA-ACY before and after HPH. (A) Adsorbed protein concentration. (B) Emulsifying activity index and emulsifying stability index. (C) Zeta potential. (D) Surface hydrophobicity.

To elucidate the mechanism by which HPH and lipid composition of oil phase influence emulsion capabilities, a series of interfacial characteristics, including AP, emulsifying properties, zeta potential, and surface hydrophobicity, were investigated. As shown in Fig. 4A and B, emulsion-tuna oil demonstrated the highest AP (95.85 g/100 g) and EAI (4.71 m^2^/g) following HPH. This may be attributed to HPH increasing the total surface area at the O—W interface within the emulsion, thereby promoting more proteins for adsorption to stabilize the interface (Lin et al., 2025). This ultimately led to an elevated EAI of emulsion. Moreover, Fan et al. (2021) noted that EAI can be summarized as the relative surface coverage of protein molecules on the individual oil droplets. Furthermore, the zeta potential of emulsion was increased from 15.60 to 18.53 mV by HPH treatment (Fig. 4C), indicating enhanced inter-droplet repulsion and improved stability of emulsion system. This trend aligns with the ESI results. Surface hydrophobicity also exhibited the same trend (Fig. 4D). This could be explained by the considerable cavitation effect induced by HPH treatment, which loosened the structure of gelatin and increased the exposure of tryptophan residues embedded within it (Gao et al., 2025). Meanwhile, the structural relaxation of the protein and its enhanced surface hydrophobicity facilitated rapid reorganization at the O—W interface, enabling the gelatin to quickly penetrate the oil phase and efficiently cover the surface of the newly formed oil droplets (Zhang et al., 2024). Finally, this contributed to the observed enhancement in both AP and EAI.

As a result of the competitive adsorption between DAG and gelatin at the O—W interface, subsequent alterations in the displacement of gelatin occurred, resulting in significantly reduced protein adsorption at the interface of emulsion-CALA-ACY in comparison to that of emulsion-tuna oil (*p* < 0.05). Chen, Lan, et al. (2023) reported similar findings, observing that competitive adsorption between DAG and soy protein isolate at the O—W interface led to lower interfacial protein concentrations in DAG-based emulsions compared to TAG-based emulsions. Moreover, the reduction of positively charged gelatin diminished the overall surface charge of emulsion. Simultaneously, DAG–gelatin interactions altered protein conformation, revealing more hydrophobic groups and enhancing surface hydrophobicity of emulsion. Notably, HPH treatment dramatically modified interfacial properties of emulsions with diverse oil phase compositions. The particle size of emulsion-CALA-ACY was abruptly reduced from 521.70 nm to 228.57 nm under the HPH treatment. On one hand, smaller droplet size conferred droplet particles with increased interfacial area, hence offering more adsorption space for DAG and gelatin (Han et al., 2024). On the other hand, DAG demonstrated superior efficiency in reducing interfacial free energy compared to protein–surfactant complexes or proteins alone (Liu et al., 2024). Ultimately, AP, EAI, and zeta potential of emulsion all exhibited substantial reductions (*p* < 0.05). In addition, the substantial adsorption of DAG and smaller particle size contributed to enhanced short-term stability of emulsion against flocculation, leading to a slightly increased ESI (Xu et al., 2022).

### 3.4 Conformation of interfacial protein in various emulsions

**Fig. 5:**
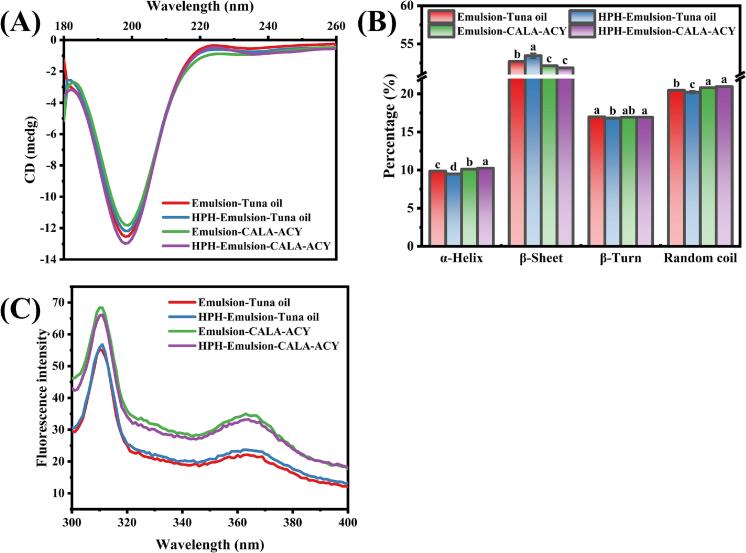
Protein conformation analysis for emulsions of tuna oil and CALA-ACY before and after HPH. (A) Secondary structure tested by Circular dichroism. (B) Relative content of protein secondary structure. (C) Tertiary structure tested by Fluorescence spectrometer.

The CD spectrum of all samples is illustrated in Fig. 5A, were characterized by a negative peak at approximately 196 nm. This is ascribed to the helical structure of collagen (type I) (Raja et al., 2016). The secondary structures' relative content of gelatin within the emulsions is shown in Fig. 5B. Following HPH treatment, emulsion-tuna oil demonstrated reduced α-helical and random coil contents, coupled with an increased β-sheet content in gelatin (*p* < 0.05). This probably because the significant energy input during HPH, compelling gelatin molecules to experience maximal unfolding, disruption, rearrangement, and reassembly (Ma et al., 2023). Eventually, a more organized protein aggregation morphology developed. This structural transition from a compact α-helical to a more extended β-sheet exposed additional hydrophobic residues, thereby promoting insertion of the protein into the oil phase and enhancing its adsorption at the O—W interface (Zhang et al., 2024). Consequently, this conformational change may also account for the significant increases in AP and EAI.

Upon the transition of oil phase from tuna oil to CALA-ACY, the β-sheet content of gelatin markedly decreased while the random coil content increased, indicating that an unfolding of gelatin structure and an evolution toward disorder. This phenomenon can be explained by the interaction between DAG and gelatin. Moreover, Wang et al. (2014) noted that the β-sheet structure in proteins is a secondary structural unit maintained by hydrogen bonds, primarily serving to stably enclose hydrophobic amino acid residues within the protein interior. Therefore, when a mixed interface film was via co-adsorption of gelatin and DAG at the interface, the intervention of DAG may disturb the secondary structure of gelatin, promoting its transformation to a more flexible and relaxed state. With the dissociation or disruption of this local structure, these typically embedded hydrophobic amino acids may become exposed externally (Wu et al., 2023). Hence, altering the interface properties of emulsion were changed. Following HPH treatment of emulsion-CALA-ACY, this trend toward unfolding and disorder appeared to be slightly more evident, suggesting that mechanical forces may further unravel gelatin chains and potentially facilitate DAG-dominated interfacial adsorption.

As shown in Fig. 5C, the fluorescence intensity of CALA-ACY-based emulsions exhibited a significantly enhanced phenomenon compared to the tuna oil-based emulsions. This may be attributed to the interaction between DAG and gelatin, which modified the microenvironment on the surface of protein molecules, triggering the unfolding of their spatial conformation and thereby exposing additional hydrophobic amino acid residues from the interior (Li, Wang, et al., 2025). Similar results were observed in the research conducted by Luo et al. (2025), they found that emulsions with elevated DAG content in the oil phase exhibited enhanced fluorescence intensity under specific pH conditions. However, HPH treatment had no significant effect on the fluorescence intensity, probably due to the insensitivity of the tertiary structure of emulsion proteins to HPH.

### 3.5 Rheological properties of various emulsions

**Fig. 6:**
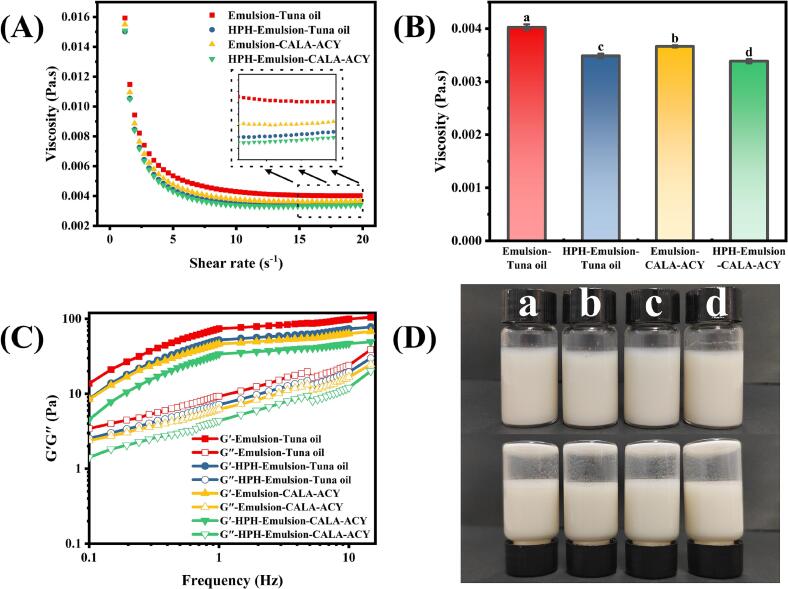
Rheological properties of emulsions prepared by tuna oil and CALA-ACY before and after HPH tested by Rheometer. (A) Apparent viscosity. (B) Specific constant value of apparent viscosity. (C) Storage modulus (G′) and loss modulus (G″) of emulsions through frequency sweeps. (D) Visual appearance of emulsions.

The effects of HPH treatment and lipid composition of oil phases on the apparent viscosity of emulsions are displayed in Fig. 6A. All emulsions demonstrated a consistent trend of an initial sharp decline in apparent viscosity followed by a gradual plateau within the designated shear rate range. This corresponds with typical shear thinning characteristics, indicating a non-Newtonian fluid. This is due to the external shear weakened inter-droplet physical interactions and disrupted the network structure within the emulsion (Xu et al., 2025). The specific constant values following shear cessation are displayed in Fig. 6B. The apparent viscosity of emulsions was significantly reduced by HPH treatment. Additionally, under consistent homogenization conditions, the apparent viscosity of CALA-ACY-based emulsions consistently remained lower than that of tuna oil-based emulsions. This phenomenon strongly associated with the reduced droplet size of emulsion. Furthermore, Liu, Ma, et al. (2025) found that smaller emulsion particle size decreased the flow resistance by enhancing the interfacial contact area between oil and continuous phases, thereby effectively decreasing the apparent viscosity.

The viscoelastic behavior of emulsions is depicted in Fig. 6C, G′ consistently exceeded G″ within the fixed-frequency scanning range of 0.1–15 Hz, signifying a weakly gelled networked structure predominantly characterized by elasticity. Similar to apparent viscosity, both HPH treatment and enzymatic hydrolysis of oil phase impaired the mechanical properties of emulsions. HPH-emulsion-CALA-ACY exhibited the minimal G′ and G″ values. This may result from reduced protein adsorption at the O—W interface (Wang et al., 2025). Notably, G″ gradually approached G′ with increasing frequency, revealing a transition toward a viscous-dominated state and macroscopic fluidic behavior (Feng et al., 2025). This is validated in Fig. 6D, where all emulsions maintained a high-flow state in the inverted vial experiment.

### 3.6 CI of different emulsions

**Fig. 7:**
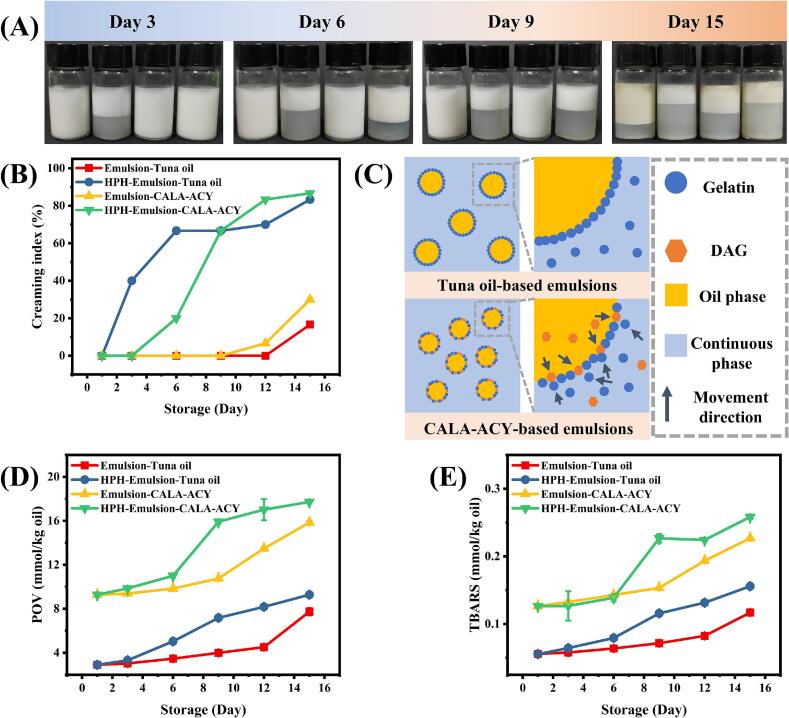
(A) Visual appearance of various emulsions after storage for 3, 6, 9, and 15 days. (B) Creaming index of emulsions formed by tuna oil and CALA-ACY before and after HPH. (C) Schematic illustration of interfacial structure of emulsions with distinct lipid compositions. (D) and (E) are peroxide value (POV) and thiobarbituric acid reactive substances (TBARS) of emulsions during storage, respectively.

The comprehensive creaming phenomenon of four emulsions formed from tuna oil and its hydrolyzed acylglycerol over a 15-day storage period is shown in Fig. 7A. The specific CI for all samples was as follows (Fig. 7B): HPH-emulsion-CALA-ACY > HPH-emulsion-tuna oil > emulsion-CALA-ACY > emulsion-tuna oil. CI represents an emulsion's capacity to separate clear liquid over time and serves as an indicator of emulsion stability, with its numerical value inversely related to the storage stability of emulsion (Liu et al., 2023). At the uniform homogenization conditions, emulsions with CALA-ACY as the oil phase exhibited higher CI than those based on tuna oil after 12 days of storage. This occurrence may due to the adsorption of DAG at the interfacial layer could not provide sufficient steric repulsion to maintain long-term droplet stability, leading to the phase separation (Cai et al., 2023). (Han et al., 2024) reported that HPH treatment decreased droplet size while concurrently substituted interfacial proteins with polyglycerol fatty acid esters, hence compromising interfacial membrane integrity. This facilitated fat crystal penetration across the interface and triggered partial coalescence. Fig. 7C illustrates the interfacial structure of emulsions with distinct lipid compositions. Thus, the modified interfacial structure observed in this study likely represents a key mechanism for emulsion separation during storage. Additionally, the CI of HPH-treated emulsions were significantly higher than those of untreated emulsions. This discrepancy likely due to the differences in viscosity. Increased viscosity reduces the droplet migration rates and collision frequency (Xiao et al., 2025). Conversely, reduced viscosity of emulsion enhanced molecular movement among droplets within the system, accelerating separation. This finding aligns with the results in Fig. 6A and B, providing mutual validation.

The formation dynamics of lipid oxidation products, including POV and TBARS, in the emulsions during storage are illustrated in Fig. 7D and E. All emulsions exhibited a progressive increase in the levels of these two oxidation product indicators with the extension of storage time, reflecting a gradual decline in emulsion stability and a continuous aggravation of lipid oxidation. Interestingly, the final POV and TBARS values of the emulsions at the end of storage were consistent with their CI. This phenomenon could be attributed to the damage of the protective layer on the surface of oil droplets caused by emulsion destabilization, which rendered the oil phase more susceptible to external light and oxygen, thereby accelerating the oxidation process. Consistently, a previous study conducted by Li et al. (2026) confirmed that the overall oxidative stability of fish oil emulsions was largely dependent on the physical interfacial barrier formed by anthocyanin–whey protein non-covalent complexes on the surface of oil droplets.

## 4 Conclusions

This paper provided a systematic investigation of the combined effects of HPH and enzymatic hydrolysis of the oil phase on the interfacial properties, protein conformation, and stability of gelatin-stabilized emulsions. HPH treatment altered the structure of interfacial proteins, as evidenced by an elevation in β-sheet content alongside a reduction in random coil content, accompanied by lower apparent viscosity but higher zeta potential. Despite these changes, the resulting emulsion exhibited reduced storage stability. Hydrolysis of tuna oil by lipase CALA produced DAGs that facilitated protein unfolding by lowering interfacial tension and competing with gelatin for adsorption. This led to a decline in β-sheet content accompanied by an increase in random coil content, demonstrating an effect opposite to that of HPH treatment. Consequently, emulsions prepared with hydrolyzed oil showed accelerated phase separation during storage due to reduced zeta potential and apparent viscosity. The results indicate that both homogenization conditions and the lipid composition of the oil phase jointly govern the interfacial behavior and stability of emulsions. Although HPH enhanced DAG adsorption at the O—W interface, further optimization of particle size in the HPH–emulsion-CALA-ACY system simultaneously reduced interfacial protein adsorption, surface charge, and viscosity, ultimately compromising emulsion stability. Considering these limitations, future research should explore the incorporation of polysaccharides to improve stability and develop stable nanoemulsion systems with high ω-3 PUFA content. Overall, the findings provide a foundation for tailored processing strategies of fish oil-based emulsions and their potential application in functional food ingredients.

## CRediT authorship contribution statement

**Junyong Xuan:** Writing – original draft, Methodology, Investigation, Formal analysis, Data curation. **Imad Khan:** Writing – review & editing. **Huilan Zeng:** Investigation, Data curation. **Zehua Qiu:** Investigation, Data curation. **Zongyuan Han:** Validation, Resources. **Zefu Wang:** Visualization, Software. **Shucheng Liu:** Supervision, Conceptualization. **Qiuyu Xia:** Writing – review & editing, Supervision, Project administration, Funding acquisition, Conceptualization.

## Declaration of competing interest

The authors declare that they have no known competing financial interests or personal relationships that could have appeared to influence the work reported in this paper.

## Data Availability

Data will be made available on request.
